# The pseudogene derived long noncoding RNA DUXAP8 promotes gastric cancer cell proliferation and migration via epigenetically silencing PLEKHO1 expression

**DOI:** 10.18632/oncotarget.11075

**Published:** 2016-08-05

**Authors:** Hong-wei Ma, Min Xie, Ming Sun, Tian-yu Chen, Rong-rong Jin, Tian-shi Ma, Qin-nan Chen, Er-bao Zhang, Xue-zhi He, Wei De, Zhi-hong Zhang

**Affiliations:** ^1^ Department of Pathology, The First Affiliated Hospital of Nanjing Medical University, Nanjing, People’s Republic of China; ^2^ Department of Biochemistry and Molecular Biology, Nanjing Medical University, Nanjing, People’s Republic of China; ^3^ Department of Oncology, Second Affiliated Hospital, Nanjing Medical University, Nanjing, People’s Republic of China; ^4^ Center for Reproduction and Genetics, Suzhou Municipal Hospital, Nanjing Medical University Affiliated Suzhou Hospital, Suzhou, People’s Republic of China

**Keywords:** gastric cancer, pseudogene, lncRNA, DUXAP8, PLEKHO1

## Abstract

Gastric cancer (GC) is the third leading cause of cancer death due to its poor prognosis and limited treatment options. Evidence indicates that pseudogene-derived long noncoding RNAs (lncRNAs) may be important players in human cancer progression, including GC. In this paper, we report that a newly discovered pseudogene-derived lncRNA named DUXAP8, a 2107-bp RNA, was remarkably upregulated in GC. Additionally, a higher level of DUXAP8 expression in GC was significantly associated with greater tumor size, advanced clinical stage, and lymphatic metastasis. Patients with a higher level of DUXAP8 expression had a relatively poor prognosis. Further experiments revealed that knockdown of DUXAP8 significantly inhibited cell proliferation and migration, as documented in the SGC7901 and BGC823 cell lines. Furthermore, RNA immunoprecipitation and chromatin immunoprecipitation assays demonstrated that DUXAP8 could epigenetically suppress the expression of PLEKHO1 by binding to EZH2 and SUZ12 (two key components of PRC2), thus promoting GC development. Taken together, our findings suggest that the pseudogene-derived lncRNA DUXAP8 promotes the progression of GC and is a potential therapeutic target for GC intervention.

## INTRODUCTION

Gastric cancer (GC) is the fifth most common cancer and third leading cause of cancer death globally, being particularly prevalent in Asia [1]. Unfortunately, more than half of patients are diagnosed at an advanced stage, which is passed the optimal time for a radical operation [2–4]. Although great progress has been made in research on GC, the basic molecular mechanisms behind its development are still poorly understood. Therefore, there remains an urgent need to decipher the different mechanisms involved in GC progression. Recently, many oncogenes and tumor suppressors have been identified as key players in GC tumorigenesis and development; however, no appropriate molecular biomarkers have been established to facilitate the comprehensive management of patients, for example, via prognostic prediction [5]. Thus, the exploration of new indicators of GC diagnosis and novel treatment targets is increasingly important.

With the development of whole-genome sequencing technology, it has gradually been elucidated that protein-coding genes constitute only 2% of the human genome, while the remainder are noncoding genes including microRNA genes, long noncoding RNA (lncRNA) genes, and pseudogenes [6]. Pseudogenes are defined as genomic loci that resemble real genes, but were considered biologically inconsequential because they harbor premature stop codons, deletions/insertions, and frameshift mutations that prevent their translation into functional proteins [7]. However, despite pseudogenes being considered as nonfunctional genomic fossils following their discovery in 1977 [8], recent studies have revealed the multilayered biological function of some pseudogenes in multiple cellular processes [9], especially their involvement in human diseases including cancers [10–13]. LncRNAs, which are defined as RNAs of more than 200 nucleotides in length and with limited protein-coding potential [14, 15], play roles in regulating a wide range of biological processes, such as cell differentiation, proliferation, apoptosis, and migration [16–18]. Recently, substantial evidence has demonstrated that pseudogene-derived lncRNAs are crucial regulators of GC development and progression. For example, pseudogene-expressed POU5F1B is amplified and expressed at a high level in GC, and its amplification is associated with a poor prognosis in GC patients [19]. In addition, the pseudogene-expressed lncRNA SUMO1P3 was found to be significantly upregulated in GC, and its expression level was significantly correlated with tumor size, differentiation, lymphatic metastasis, and invasion [20]. Therefore, pseudogenes have been highlighted as key fators in cancer research.

The pleckstrin homology domain-containing protein casein kinase-2 interacting protein-1 (PLEKHO1; also known as CKIP-1) was originally reported to interact specifically with the casein kinase-2 (CK2) α-subunit but not the α-subunit [21]. Subsequently, a number of studies indicated that PLEKHO1 is a scaffold protein that mediates interactions with multiple proteins. In addition, PLEKHO1 was found to interact with Akt and inhibit Akt kinase activity [22]. It was also revealed to suppress fibrosarcoma cell survival, possibly by downregulating phosphatidylinositol-3-kinase (PI3K)/Akt signaling. Finally, the growth of stable PLEKHO1 transfectants xenografted into nude mice was also slower than that of mock transfectants. These findings suggest that PLEKHO1 is a candidate tumor suppressor with Akt inhibitory function [22].

Given the importance of pseudogenes in GC, in the current study, we showed that DUXAP8, a 2107-bp RNA, was remarkably upregulated in GC tissues compared with that in corresponding nontumor tissues. We further discovered that DUXAP8 upregulation was also correlated with larger tumor size, advanced clinical stage, lymphatic metastasis, and poor prognosis of patients with GC. Moreover, functional analysis indicated that DUXAP8 promoted GC cell growth both *in vitro* and *in vivo* by epigenetically silencing PLEKHO1 transcription via binding to EZH2 and SUZ12. These results suggest that DUXAP8 may act as a noncoding oncogene in GC tumorigenesis and is a potential biomarker for GC diagnosis and gene therapy.

## RESULTS

### DUXAP8 expression is upregulated in human GC tissues

**Figure 1:**
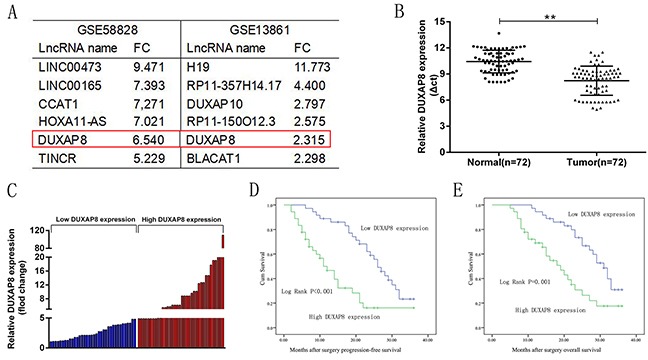
Relative DUXAP8 expression in GC tissues and its clinical significance **A**. Relative expression of DUXAP8 in human gastric cancerous tissues compared with noncancerous tissue via GSE58828 and GSE13861 data analysis. **B**. Relative expression of DUXAP8 in human GC tissues (n = 72) compared with corresponding non-tumor tissues (n= 72). DUXAP8 expression was examined by qRT-PCR and normalized to GAPDH expression (shown as Δct). **C**. Results are presented as the fold-change in tumor tissues relative to normal tissues, and DUXAP8 expression was classifid into two groups. **D, E**. Kaplan-Meier progression-free survival and overall survival curves according to DUXAP8 expression level. Error bars indicate mean ± standard errors of the mean. *P < 0.05, **P < 0.01.

In this study, we initially analyzed the expression level of the pseudogene-derived lncRNA DUXAP8 in human GC tissues by using microarray data downloaded from the Gene Expression Omnibus (GEO; GSE58828[23] and GSE13861[24]), and found that the DUXAP8 expression level was significantly upregulated in GC tissues compared with that in normal tissues (Figure 1A). Furthermore, the expression level of DUXAP8 in 72 paired GC tissues and adjacent normal tissues was determined by qRT-PCR and normalized to GAPDH. Similarly, this showed that DUXAP8 expression was significantly upregulated in GC tissues compared with that in their normal counterparts (P<0.01) (Figure 1B).

### Overexpression of DUXAP8 is associated with TNM stage, tumor size, lymphatic metastasis and poor prognosis of GC

To assess the correlation between DUXAP8 expression and clinicopathological features, the 72 primary GC patients were classified into two groups relative to the median ratio of DUXAP8 expression in tumor tissues: a high-DUXAP8 group (n=36, DUXAP8 expression ratio ≥ median) and a low-DUXAP8 group (n=36, DUXAP8 expression ratio < median ratio) (Figure 1C). The clinicopathological characteristics of the 72 primary GC patients were summarized in Table 1. Noticeably, high DUXAP8 expression in GC was significantly correlated with advanced TNM stage (P=0.001), lymph node metastasis (P=0.007), and tumor size (P=0.002). However, DUXAP8 expression was not associated with other parameters such as gender (P=0.635) and age (P=0.157) in GC (Table 1). To determine the relationship between DUXAP8 expression and the prognosis of GC patients after gastrectomy, progression-free survival (PFS) and overall survival (OS) curves were plotted according to DUXAP8 expression level and analyzed by the Kaplan–Meier method and log-rank test, respectively (Figure 1D and 1E). The results showed that the PFS rate over 3 years for cases with high DUXAP8 expression was 27.8%, while it was 33.3% for low DUXAP8 expression. The median survival time for cases with high DUXAP8 expression was 11 months, while it was 26 months for low DUXAP8 expression (Figure 1D, log-rank P<0.001). Moreover, the overall survival rate over 3 years for cases with high DUXAP8 expression was 30.6%, but 41.7% for low DUXAP8 expression. Finally, the median survival time for cases with high DUXAP8 expression was 19 months, but 31 months for low DUXAP8 expression (Figure 1E, log rank P=0.001). These results indicate that DUXAP8 may be a useful marker of the prognosis or progression of GC.

**Table 1:** Correlation between DUXAP8 expression and clinicopathological characteristics of gastric cancer patients

Characteristics	N(%)	DUXAP8		P Chi-squared test P-value
		High NO. cases (36)	Low NO. cases (36)	
**Gender**				0.635
Male	40(55.6%)	21	19	
Female	32(44.4%)	15	17	
**Age**				0.157
≤65	34(47.2%)	14	20	
>65	38(52.8%)	22	16	
**Histological subtype**				0.339
Squamous cell carcinoma	30(41.7%)	13	17	
Adenocarcinoma	42(58.3%)	23	19	
**Stage**				0.001*
I	22(30.6%)	5	17	
II	24(33.3%)	11	13	
III	26(36.1%)	20	6	
**Lymph node metastasis**				0.007*
Negative	27(37.5%)	8	19	
Positive	45(62.5%)	28	17	
**Tumor size**				0.002*
≤5 cm	31(43.1%)	9	22	
>5 cm	41(56.9%)	27	14	
**HP infection**				0.471
Negative	29(40.3%)	13	16	
Positive	43(59.7%)	23	20	

*P<0.05 was considered significant

### DUXAP8 promotes GC cell proliferation *in vitro*

**Figure 2:**
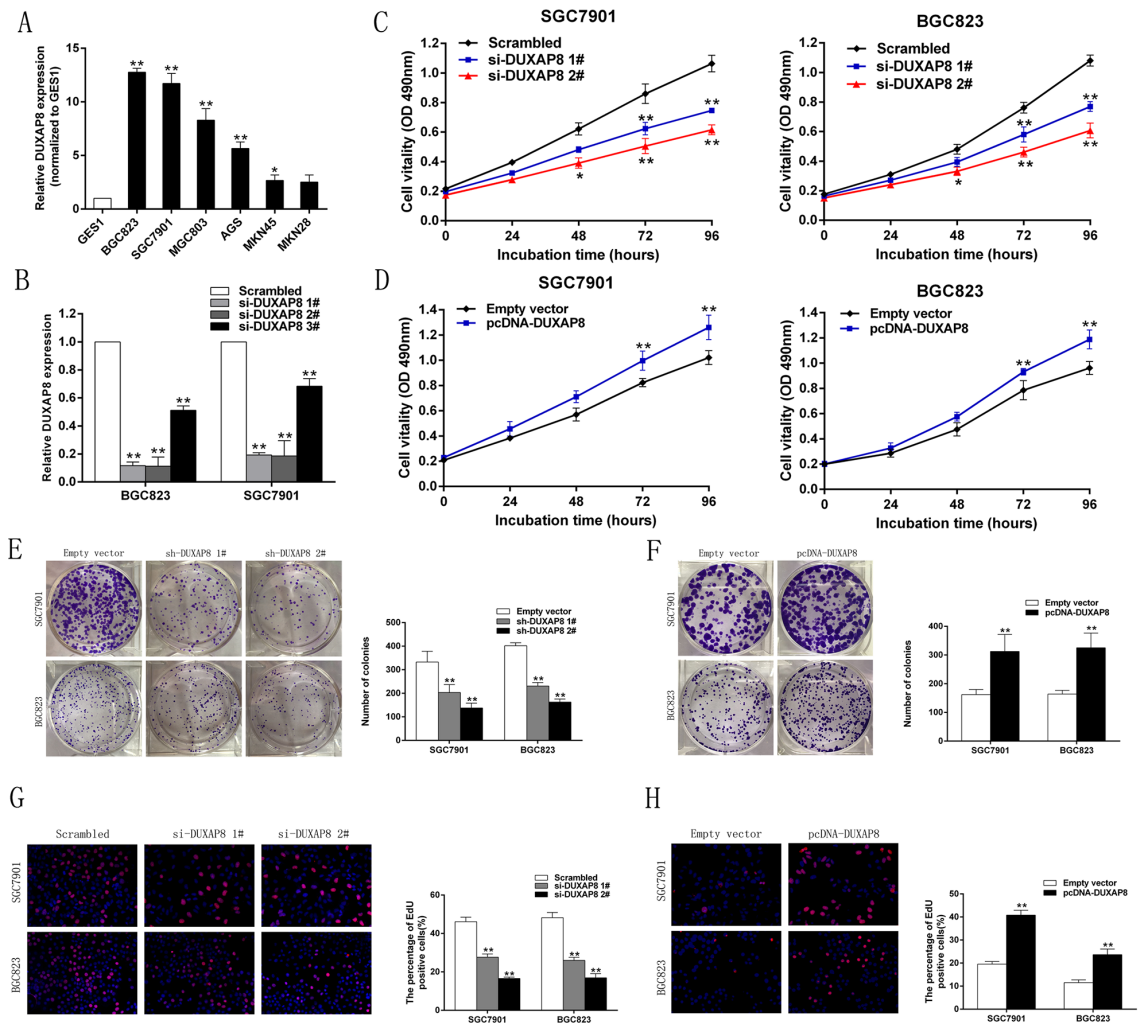
DUXAP8 promotes GC cell proliferation *in vitro* **A**. QRT-PCR analysis of DUXAP8 expression in the normal gastric epithelium cell line (GES1) and GC cells. **B**. QRT-PCR analysis of DUXAP8 expression in control (scrambled), si-DUXAP8 1#, si-DUXAP8 2# and si-DUXAP8 3# treated GC cells. **C, D**. MTT assays were used to determine the viability of si-DUXAP8-transfected or pcDNA-DUXAP8-transfected GC cells. Experiments were performed in triplicate. **E, F**. Colony formation assays were performed to determine the proliferation of sh-DUXAP8-transfected or pcDNA-DUXAP8- transfected GC cells. Colonies were counted and captured. **G, H**. Proliferating SGC7901 and BGC823 cells were labeled with Edu. The Click-it reaction revealed Edu staining (red). Cell nuclei were stained with DAPI (blue). Representative images and data based on three independent experiments. Error bars indicate mean ± standard errors of the mean. *P < 0.05, **P < 0.01.

To investigate the functional role of DUXAP8 in GC cells, we first performed qRT-PCR analysis to detect its expression in diverse human GC cell lines. As shown in Figure 2A, DUXAP8 expression was significantly upregulated in two GC cell lines (SGC7901 and BGC823) compared with that in the normal gastric epithelium cell line (GES-1). Next, we designed three different DUXAP8 siRNAs for transfection into the SGC7901 and BGC823 cell lines. qRT-PCR analysis was performed 48 h post-transfection and the data revealed that all of the DUXAP8 siRNAs effectively entered the cells, in addition, si-DUXAP8 1# and 2# exhibited more efficient interference than si-DUXAP8 3# (Figure 2B). Therefore, we selected si-DUXAP8 1# and 2# for use in the subsequent experiments. Meanwhile, we also induced the ectopic overexpression of DUXAP8 by transfecting GC cell lines with a pcDNA-DUXAP8 expression vector. Here, we assessed the expression of DUXAP8 by qRT-PCR; it was found to increase significantly in the pcDNA-DUXAP8-transfected cells compared with those transfected with the empty vector (Supplementary Figure S1A).

MTT assays showed that knockdown of DUXAP8 expression significantly inhibited the growth of SGC7901 and BGC823 cells compared with the use of the corresponding scrambled control (Figure 2C). In contrast, DUXAP8 overexpression promoted the growth of GC cells (Figure 2D). Similarly, the results of colony-formation assays revealed that clonogenic survival was significantly decreased following downregulation of DUXAP8 in SGC7901 and BGC823 cells (Figure 2E), but markedly increased due to the overexpression of DUXAP8 (Figure 2F). Ethynyl deoxyuridine (EdU) (red)/DAPI (blue) immunostaining also confirmed this result; knockdown of DUXAP8 expression significantly decreased the rate of proliferating cells, while its overexpression had the opposite effect (Figure 2G and 2H). These findings indicate that DUXAP8 may act as an oncogene involved in the promotion of GC cell proliferation.

### Downregulation of DUXAP8 induces apoptosis of GC cells

**Figure 3:**
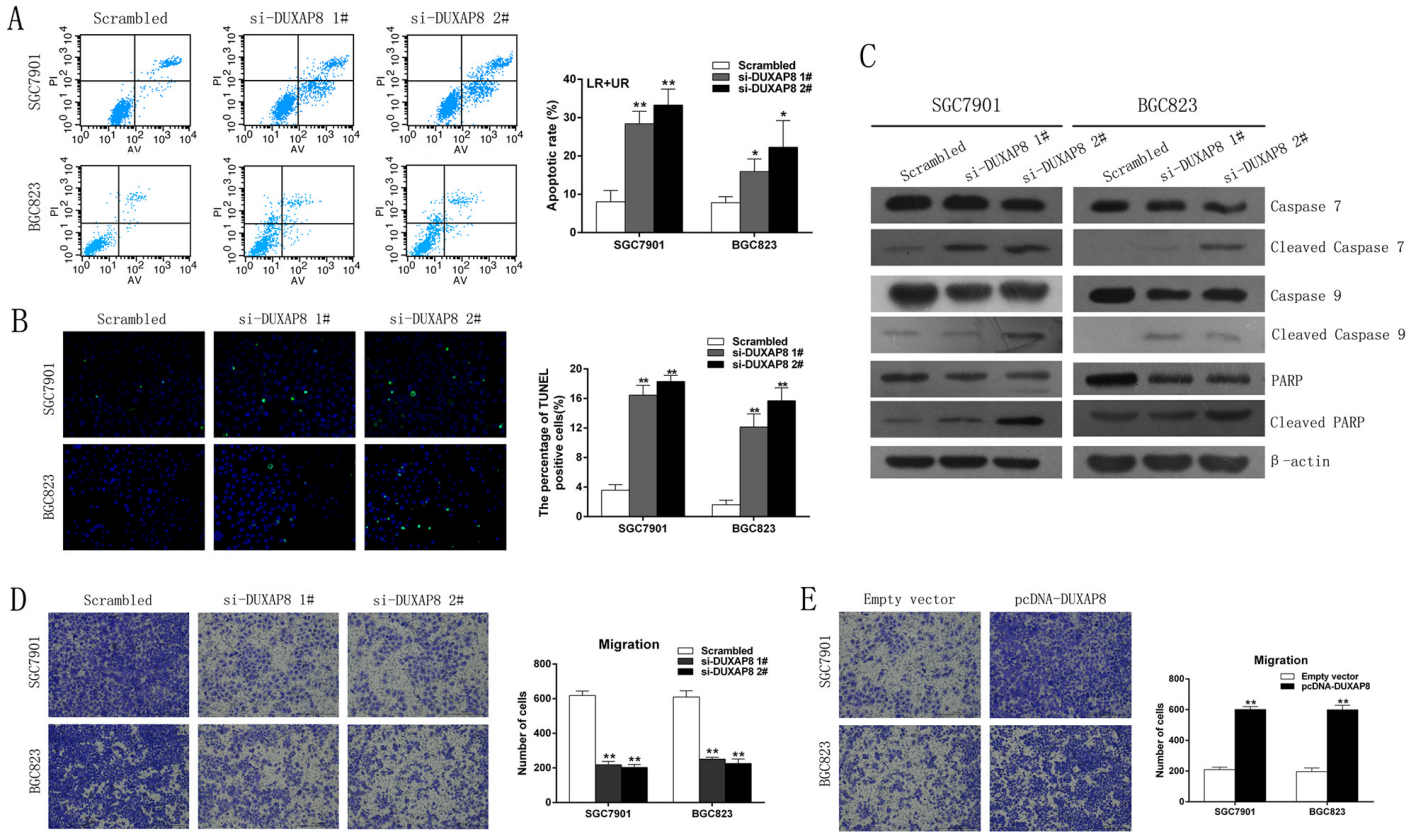
Effect of DUXAP8 on GC cell apoptosis and migration*in vitro* **A**. Flow cytometry was used to detect the apoptotic rates of cells. LR, early apoptotic cells; UR, terminal apoptotic cells. **B**. Apoptosis in SGC7901 and BGC823 cells after DUXAP8 knockdown was detected through TUNEL staining. **C**. Western blot analysis of apoptosis - related proteins after scrambled siRNA, si-DUXAP8 1#, or si-DUXAP8 2# transfection in SGC7901 and BGC823 cells. β-actin protein was used as an internal control. **D, E**. Transwell assays were performed to investigate the changes in migratory abilities of GC cells. Error bars indicate mean ± standard errors of the mean. *P < 0.05, **P < 0.01.

The level of apoptosis and cell cycle regulation were identified as two factors that contribute to GC cell growth, so we performed flow-cytometric analysis to characterize these factors. The results of cell apoptosis revealed that the proportion of apoptotic cells following the DUXAP8 siRNAs treatment was significantly increased compared with the scrambled control (Figure 3A). However, the results for the cell cycle showed no statistically significant differences in the proportions of cells in different phases of the cell cycle (data not shown). Terminal deoxynucleotidyl transferase-mediated dUTP nick end labeling (TUNEL) assay showed the same results (Figure 3B). Furthermore, western blot analysis showed that the protein levels of cleaved caspase-7, cleaved caspase-9, and cleaved PARP were increased in the cells treated with DUXAP8 siRNAs, confirming that DUXAP8 is associated with apoptosis (Figure 3C). Taken together, these results indicate that DUXAP8 may drive GC cell proliferation by inhibiting apoptosis.

### DUXAP8 promotes GC cell migration *in vitro*

Cell migration is an important aspect of cancer progression, so we evaluated GC cell migration using a transwell assay. As shown in Figure 3D, the knockdown of DUXAP8 inhibited the migratory ability of SGC7901 and BGC823 cells, and the number of migratory cells was significantly decreased. Conversely, the overexpression of DUXAP8 promoted GC cell migration (Figure 3E). A parallel invasion assay was also performed, but the results were not statistically significant (data not shown). These results imply that DUXAP8 has oncogenic properties that can promote the migration of GC cells.

### DUXAP8 epigenetically silences PLEKHO1 transcription by binding with PRC2

**Figure 4:**
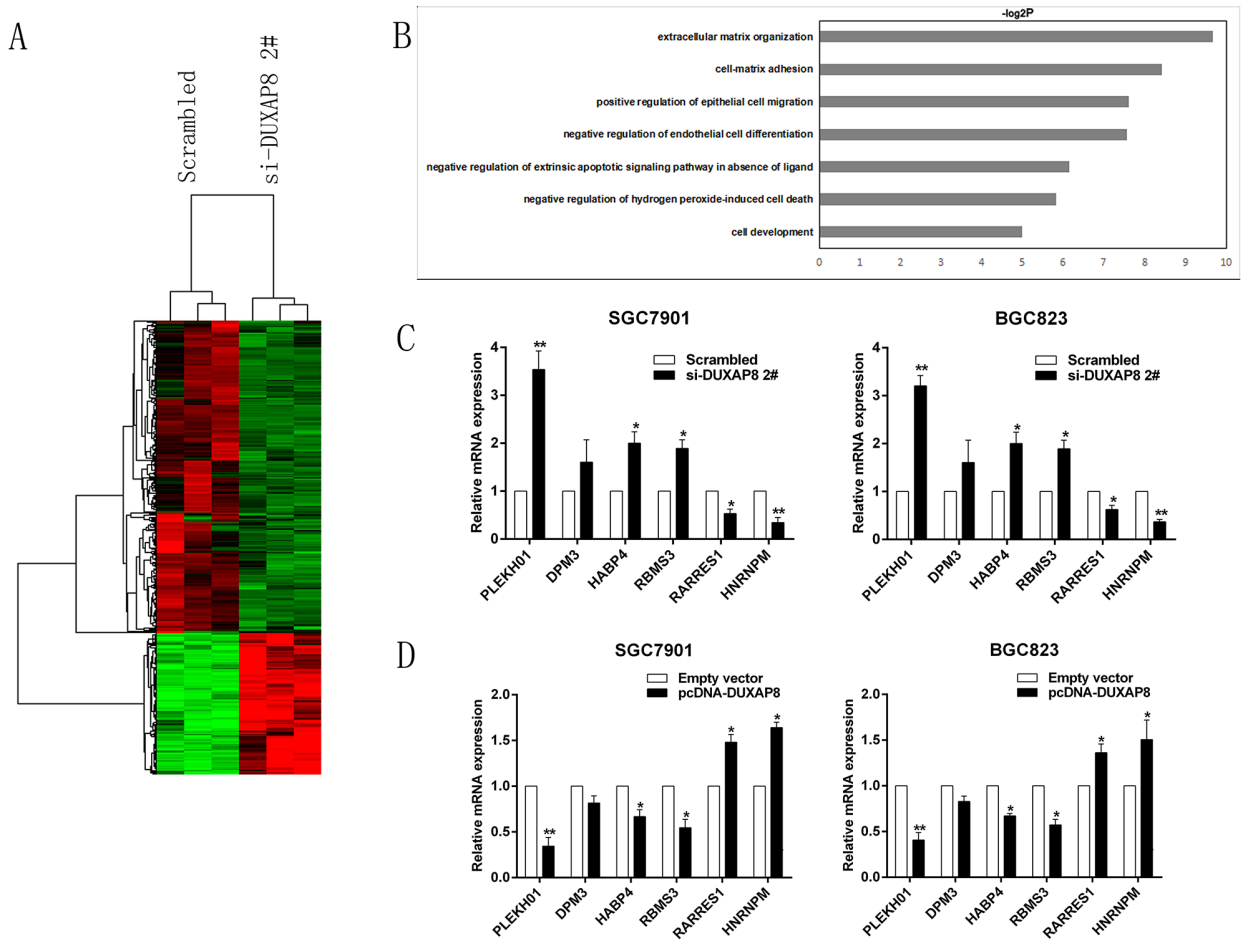
DUXAP8 knockdown increases the expression of genes involved in cell proliferation and migration **A**. Mean-centered, hierarchical clustering of 448 transcripts altered in scrambled siRNA-treated cells and si-DUXAP8-treated cells, with three repeats. **B**. Gene Ontology analysis for all genes with altered expressions between the scrambled siRNA-treated and si-DUXAP8-treated cells *in vitro*. Cell apoptosis and migration were both among the significant biological processes for genes whose transcripts level were changed in the DUXAP8-depleted GC cells. **C, D**. QRT-PCR analysis in si-DUXAP8- treated or pcDNA-DUXAP8-treated GC cells reveal altered mRNA level of genes involved in cell proliferation and migration upon DUXAP8 depletion. Error bars indicate mean ± standard errors of the mean. *P < 0.05, **P < 0.01.

To obtain unbiased findings on the DUXAP8-associated pathway, we assessed the gene expression profiles of GC cells in which DUXAP8 expression was suppressed. Specifically, we performed RNA transcriptome sequencing from control and DUXAP8-depleted BGC823 cells. BGC823 cells were treated with a scrambled siRNA or si-DUXAP8 2# for 48 h. Analysis of the RNA transcriptome sequencing data from triplicate samples revealed that a common set of 133 mRNAs exhibited increased expression in DUXAP8-depleted cells, while 315 mRNAs were downregulated (Supplementary Table S1; Figure 4A). To further study the involved pathways activated by DUXAP8, we analyzed the associated genes using data collected from the Gene Ontology (GO) database. The most prominent GO categories were related to apoptosis and cell migration, suggesting that these biological processes are particularly affected in DUXAP8-knockdown cells (Figure 4B). The GO results were essentially in agreement with our experimental findings. qRT-PCR was then used to confirm the changes in the levels of several upregulated or downregulated mRNAs involved in cell proliferation, apoptosis, and migration. The results showed that knockdown of DUXAP8 increased PLEKHO1, DPM3, HABP4, and RBMS3 expression, but decreased RARRES1 and HNRNPM` expression (Figure 4C). In support of this, the overexpression of DUXAP8 had the opposite results (Figure 4D). These findings indicate that the dysregulated genes may be key downstream mediators of DUXAP8.

**Figure 5:**
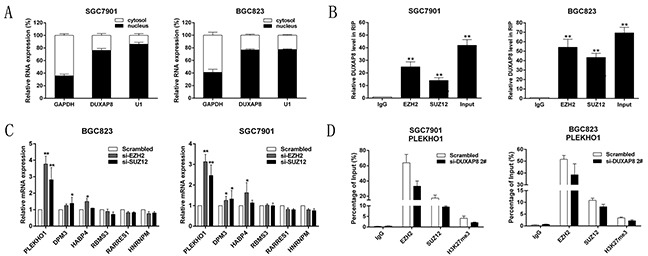
DUXAP8 epigenetically silences PLEKHO1 transcription by binding with PRC2 **A**. DUXAP8 expression levels in cell nucleus or cytoplasm of SGC7901 and BGC823 cells were detected by qRT-PCR. U6 was used as a nucleus marker and GAPDH was used as a cytosol marker. **B**. RIP experiments were performed in SGC7901, BGC823 cells and the coprecipitated RNA was subjected to qRT-PCR for DUXAP8. The fold enrichment of DUXAP8 in EZH2/SUZ12/LSD1 RIP is relative to its matching IgG control. **C**. QRT-PCR analysis of PLEKHO1, DPM3, HABP4, RBMS3, RARRES1 and HNRNPM expression levels in control, si-EZH2 and si-SUZ12 treated GC cells. **D**. ChIp-qRT-PCR of EZH2 occupancy, SUZ12 occupancy and H3K27me3 binding in the PLEKHO1 promoters in SGC7901, BGC823 cells treated with control or si-DUXAP8 2# (48h); IgG as a negative control. Error bars indicate mean ± standard errors of the mean. *P < 0.05, **P < 0.01.

To determine the distribution of DUXAP8 in GC cells, we subjected GC cell lines to fractionation and obtained the nuclear and cytoplasmic fractions. We found that DUXAP8 RNA was mostly located in the nucleus rather than the cytosol (Figure 5A), suggesting that it exerts regulatory functions at the transcriptional level. An overabundance of GAPDH or U1 RNA was used as an indicator of successful fractionation. Recently, several studies have concluded that approximately 20% of lncRNAs can regulate downstream target genes by binding with Polycomb repressive complexe 2 (PRC2) [25]. PRC2 is a methyltransferase that trimethylates H3K27 to suppress the transcription of specific genes; two of its major components are Enhancer of zeste homolog 2 (EZH2) and Suppressor of zeste 12 homolog (SUZ12) [26]. Our previous study demonstrated that HOXA-AS2 can epigenetically silence P21/PLK3/DDIT3 expression via binding to EZH2 [27]; In addition, ANRIL was shown to be able to crosstalk with microRNAs by binding to PRC2 and thus regulate GC growth [5]. Given this background, we conducted RNA immunoprecipitation (RIP) analysis to confirm that DUXAP8 binds to PRC2. As shown in Figure 5B, endogenous DUXAP8 was enriched in the anti-EZH2 and anti-SUZ12 RIP fractions in SGC7901 and BGC823 cells. HOTAIR, a known PRC2 associated lncRNA, was used as a positive control[28] (Supplementary Figure S1B). Our findings indicate that DUXAP8 may epigenetically inhibit downstream target genes by binding to EZH2 and SUZ12.

EZH2 or SUZ12 siRNAs were transfected into SGC7901 and BGC823 cells, which effectively decreased the expression of EZH2 or SUZ12 (Supplementary Figure S1C). Next, we found that the expression of PLEKHO1, DPM3, HABP4, and RBMS3 was increased in EZH2-depleted and SUZ12-depleted GC cells (Figure 5C). Based on our qRT-PCR data (Figure 4C and 5C), PLEKHO1 was the most upregulated mRNA not only in DUXAP8-depleted GC cells, but also in EZH2-depleted and SUZ12-depleted ones. These findings together indicate that PLEKHO1 may be a key downstream gene of DUXAP8, and DUXAP8 can inhibit its expression by binding to EZH2 and SUZ12. Furthermore, the results of chromatin immunoprecipitation (ChIP) assays showed that EZH2 and SUZ12 could directly bind to PLEKHO1 promoter regions and induce the histone H3 lysine 27 trimethylation (H3K27me3) modification in SGC7901 and BGC823 cells (Supplementary Figure S1D). Knockdown of DUXAP8 resulted in reduced EZH2 binding, SUZ12 binding, and H3K27me3 occupancy of the PLEKHO1 promoter (Figure 5D). These results suggest that DUXAP8 could promote GC cell growth partly through epigenetically silencing PLEKHO1 transcription by binding to EZH2 and SUZ12.

### PLEKHO1 inhibition is potentially involved in the oncogenic function of DUXAP8

**Figure 6:**
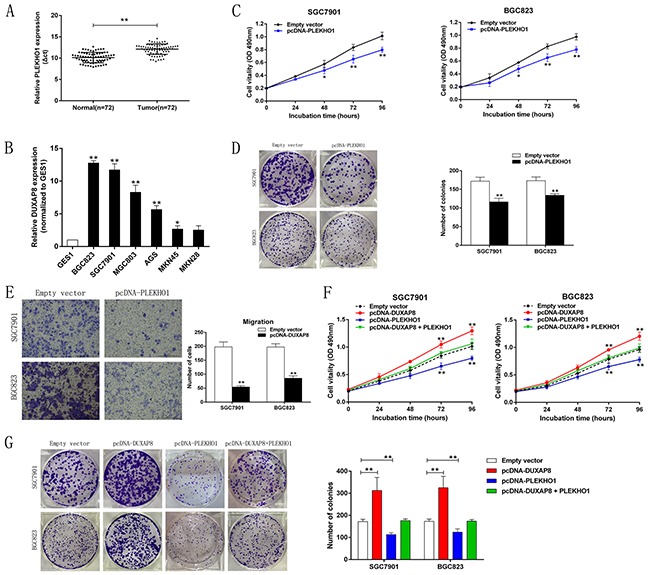
Down-regulation of PLEKHO1 promotes GC cell proliferation and is involved in the oncogene function of DUXAP8 **A**. QRT-PCR analysis of PLEKHO1 expression in 72 paired human GC tissues and adjacent noncancerous tissuesin. **B**. QRT-PCR analysis of PLEKHO1 expression in the normal gastric epithelium cell line (GES1) and GC cells. **C**. MTT assays were used to determine the cell viability for pcDNA-PLEKHO1-transfected GC cells. Experiments were performed in triplicate. **D**. Colony-formation assays were used to determine the cell proliferation for pcDNA-PLEKHO1-transfected GC cells. Experiments were performed in triplicate. **E**. Transwell assays were performed to investigate the changes in migratory abilities of GC cells. **F**. MTT assays were used to determine the cell viability for pcDNA-DUXAP8 and pcDNA-PLEKHO1 co-transfected GC cells. Experiments were performed in triplicate. **G**. Colony-formation assays were used to determine the cell viability for pcDNA-DUXAP8 and pcDNA-PLEKHO1 co-transfected GC cells. Experiments were performed in triplicate. Error bars indicate mean ± standard errors of the mean. *P < 0.05, **P < 0.01.

To assess the relationship between PLEKHO1 and DUXAP8 expression in GC, we determined the level of PLEKHO1 expression by qRT-PCR in 72 pairs of GC and matched normal tissues and several GC cell lines. The results revealed that the expression level of PLEKHO1 was remarkably reduced in GC tissues and cells compared with that in matched normal tissues and cells, respectively (Figure 6A and 6B). To confirm the influence of PLEKHO1 on the proliferation of GC cells, we induced its ectopic overexpression in SGC7901 and BGC823 cells (Supplementary Figure S1E). We then performed MTT and colony formation assays to determine cell viability. The results revealed that overexpression of PLEKHO1 inhibited the proliferation of cells (Figure 6C and 6D). We next performed transwell assays, which indicated that the overexpression of PLEKHO1 decreased GC cell migration (Figure 6E). These findings show that, similar to the results for DUXAP8 downregulation, PLEKHO1 inhibited the proliferation and migration of GC cell.

To investigate whether PLEKHO1 is involved in DUXAP8-induced GC cell proliferation, we carried out rescue experiments. Here, SGC7901 and BGC823 cells were cotransfected with pcDNA-DUXAP8 and pcDNA-PLEKHO1. MTT and colony formation assays indicated that this cotransfection partially rescued pcDNA-DUXAP8-impaired proliferation in SGC7901 and BGC823 cells (Figure 6F and 6G). These findings indicate that DUXAP8 promotes GC cell proliferation partly through downregulating PLEKHO1 expression.

### DUXAP8 promotes tumorigenesis of GC cells *in vivo*

**Figure 7:**
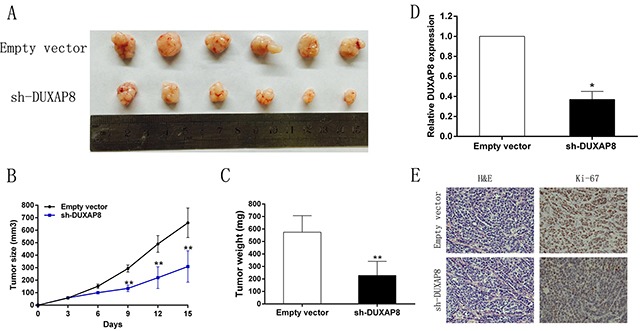
DUXAP8 promotes tumorigenesis of GC cells *in vivo* **A**. Empty vector or sh-DUXAP8 were transfected into SGC7901 cells, which were injected in the nude mice (n = 6), respectively. Tumors formed in sh-DUXAP8 group were dramatically smaller than the control group. **B**. Tumor volumes were calculated after injection every three days. Points, mean (n = 6); bars indicate SD. **C**. Tumor weights were represented as means of tumor weights ± SD. **D**. QRT-PCR was performed to detect the average expression of DUXAP8 in xenograft tumors (n = 6). **E**. The tumor sections were under H&E staining and IHC staining using antibodies against ki-67. Error bars indicate mean ± standard errors of the mean. *P < 0.05, **P < 0.01.

To determine whether the level of DUXAP8 expression affects tumorigenesis *in vivo*, sh-DUXAP8 or empty vector-transfected SGC7901 cells were inoculated into nude mice. All mice developed xenograft tumors at the injection site. Fifteen days after injection, we found that the tumors formed in the sh-DUXAP8 group were significantly smaller than those in the control group (Figure 7A). Moreover, tumor growth in the sh-DUXAP8 group was significantly slower than that in the control group (Figure 7B). Additionally, the average tumor weight was clearly lower in the sh-DUXAP8 group than in the control group (Figure 7C). qRT-PCR analysis also revealed that the level of DUXAP8 expression in tumor tissues formed from sh-DUXAP8 cells was lower than in tumors formed in the control group (Figure 7D). The tumors developed from sh-DUXAP8 cells displayed lower-intensity Ki-67 staining than the tumors formed from empty vector-transfected cells (Figure 7E). These results indicate that DUXAP8 overexpression is significantly associated with the proliferation capacity of GC cells *in vivo*.

## DISCUSSION

Pseudogenes were long considered to be nonfunctional relics littering the genome, but an increasing number of studies have emphasized their significance due to the implementation of the GENCODE project. Some studies have also highlighted the involvement of pseudogenes in the pathogenesis of diseases including cancer [29, 30]. In particular, lncRNAs have been confirmed to be involved in cancer development in humans [16–18]. Recently, more evidence has emerged that the dysregulation of pseudogene-expressed lncRNAs in GC could be one of the driving forces behind GC tumorigenesis [19, 20, 31]. Against this background, there is an urgent need to identify pseudogene-derived lncRNAs and investigate their biological functions and clinical significance. This could lead to advances in lncRNA-directed diagnosis and prognosis of this malignant disease. In this study, we ascertained that the expression of the pseudogene-derived lncRNA DUXAP8 was upregulated in GC tissues compared with that in the corresponding nontumor tissues. In addition, the upregulation of DUXAP8 was associated with a poor prognosis of GC patients, indicating that DUXAP8 may be an important clinical marker in GC therapy and prognosis. Additionally, DUXAP8 knockdown could significantly inhibit GC cell proliferation and migration.

The influence of pseudogene-derived lncRNAs in human cancer may be associated with their ability to impact on cellular functions through various mechanisms. For example, pseudogenes can act as sources of competing endogenous RNAs (ceRNAs) for microRNA sponges, RNA-binding proteins (RBPs), or translational machinery, and can also generate into endogenous small interfering RNAs [32, 33]. For instance, PTENP1 has been found to be downregulated in clear-cell renal cell carcinoma tissues and cells due to methylation, and it suppressed cancer progression by functioning as a ceRNA through acting as a decoy for miR-21 [34, 35]. In addition, Chan et al. found that PPM1KP can exert tumor suppressor activity independent of its parental gene by generation of endo-siRNAs that regulate human cell growth [36]. Moreover, the pseudogenes can recruit the histone modification protein EZH2 to target a gene promoter, thereby regulating their transcription [37]. It is evident that lncRNAs can bind to PRC2 in various cells, and silence downstream target genes. EZH2, a key catalytic subunit of PRC2, functions as a histone methyltransferase that specifically induces H3K27me3 [38, 39]. In this study, we found that DUXAP8 could epigenetically silence the transcription of downstream target genes by recruiting and binding to PRC2.

RNA transcriptome sequencing analysis indicated that PLEKHO1 may be a key downstream mediator of DUXAP8. Nie et al. found that PLEKHO1 was downregulated in various colon cancer cell lines and its level was decreased 62% in human colon cancer tissues compared with that in normal mucosal tissues. In addition, they showed that the downregulation of PLEKHO1 in colon cancers might be reversed by methylation of the promoter of the PLEKHO1 gene [40]. The findings presented here reveal that PLEKHO1 is a candidate tumor suppressor in cancer cells. In the present study, we showed for the first time that PLEKHO1 is epigenetically silenced by DUXAP8-PRC2 regulation in GC cells.

In summary, this work shows for the first time that DUXAP8 is upregulated in GC tissues and its upregulation may be associated with the poor prognosis of GC patients. DUXAP8 can promote GC cell proliferation and tumorigenesis partly through epigenetically silencing PLEKHO1 transcription by binding to PRC2. Collectively, our results provide a new perspective that the pseudogene-derived lncRNA DUXAP8 may act as a noncoding oncogene in GC tumorigenesis and could be a novel target for the early diagnosis and treatment of GC. However, the other possible mechanisms by which DUXAP8 participates in the biological functions of GC cells remain to be comprehensively determined.

## MATERIALS AND METHODS

### Differential expression analysis

GC gene expression data were downloaded from the GEO. The independent data sets from GSE58828 (http://www.ncbi.nlm.nih.gov/geo/query/acc.cgi?acc=GSE58828) and GSE13861 (http://www.ncbi.nlm.nih.gov/geo/query/acc.cgi?acc=GSE13861) were included in this study and normalized using the Robust Multichip Average. After we had downloaded probe sequences from GEO or the microarray manufacturers, Blast+2.2.30 was used to reannotate the probes in the GENCODE Release 20 sequence databases for lncRNA.

### Tissue samples and clinical data collection

A total of 72 primary GC patients who had undergone surgery at the First Affiliated Hospital of Nanjing Medical University (Nanjing, Jiangsu, China) were analyzed in this study. No local or systemic treatment had been conducted on these patients before surgery. This study was approved by the Research Ethics Committee of Nanjing Medical University and written informed consent was obtained from all patients. The 72 pairs of cancerous and corresponding adjacent nontumorous gastric tissue samples were immediately snap-frozen in liquid nitrogen and stored at −80°C until required. The clinicopathological characteristics of the GC patients are summarized in Table 1. OS was defined as the interval between the dates of surgery and death. PFS was defined as the interval between the dates of surgery and recurrence; if recurrence was not diagnosed, patients were censored at the date of death or the last follow-up.

### Cell culture

Human GC cell lines (SGC7901, BGC823) and a normal gastric epithelial cell line (GES-1) were purchased from the Institute of Biochemistry and Cell Biology of the Chinese Academy of Sciences (Shanghai, China). Cells were cultured in RPMI 1640 or Dulbecco’s Modified Eagle Medium (DMEM; GIBCO-BRL) supplemented with 10% fetal bovine serum (Gibco), 100 U/ml penicillin, and 100 mg/ml streptomycin in humidified air at 37°C with 5% CO_2_. BGC823 cells were cultured in RPMI 1640 medium and SGC7901 cells were cultured in DMEM (GIBCO-BRL) medium.

### RNA extraction and qRT-PCR analyses

Total RNA was extracted from tissues or cultured cells using TRIZOL reagent (Invitrogen). For qRT-PCR, RNA was reverse-transcribed to cDNA using a Reverse Transcription Kit (Takara, Dalian, China). Real-time PCR analyses were performed with SYBR Premix Ex Taq (Takara). Results were normalized to the expression of GAPDH. The specific primers are listed in Supplementary Table S2. The qRT-PCR analysis was conducted on an ABI 7500, and data were collected with this instrument. Our qRT-PCR results were analyzed, expressed relative to threshold cycle values, and then converted to fold changes.

### Transfection of gastric cancer cells

GC cells were transfected with siRNAs and plasmid vectors using Lipofectamine 2000 (Invitrogen, USA), in accordance with the manufacturer’s protocol. Three individual DUXAP8 siRNAs (si-DUXAP8 1#, 2#, and 3#), SUZ12 siRNA, EZH2 siRNA, and scrambled negative control siRNA (si-NC) were purchased from Invitrogen. The nucleotide sequences of siRNAs for DUXAP8, SUZ12, and EZH2 are listed in Supplementary Table S2. The full-length complementary DNA of DUXAP8 and PLEKHO1 was synthesized by Realgene (Nanjing, China) and subcloned into the pcDNA3.1(+) vector (Invitrogen), in accordance with the manufacturer’s instructions. At 48 h post-transfection, cells were harvested for qRT-PCR or western blot analysis.

### Cell proliferation assays

Cell viability was tested with Cell Proliferation Reagent Kit I (MTT) (Roche Applied Science). BGC823 and SGC7901 cells transfected with si-DUXAP8 or pcDNA-DUXAP8 (3000 cells/well) were grown on 96-well plates. Cell viability was assessed every 24 h following the manufacturer’s protocol. All experiments were performed in quadruplicate. For colony formation assays, a certain number of transfected cells were placed in each well of six-well plates and maintained in appropriate medium containing 10% fetal bovine serum for 2 weeks, during which the medium was replaced every 4 days. After 14 days, the cells were fixed with methanol and stained with 0.1% crystal violet (Sigma-Aldrich). Visible colonies were then counted. For each treatment group, wells were assessed in triplicate, and experiments were independently repeated three times.

### EdU analysis

Proliferating cells were assessed using a 5-ethynyl-2-deoxyuridine (EdU) labeling/detection kit (Ribobio, Guangzhou, China), in accordance with the manufacturer’s protocol. Briefly, breast cancer cells were cultured in 96-well plates at 5 × 10^3^ cells per well and transfected with plasmid DNA or siRNA for 48 h. Then, 50 μM EdU labeling medium was added to the cell culture and incubated for 2 h at 37°C under 5% CO_2_. Next, the cultured cells were fixed with 4% paraformaldehyde (pH 7.4) for 30 min and treated with 0.5% Triton X-100 for 20 min at room temperature. After washing with phosphate-buffered saline (PBS), the samples were stained with anti-EdU working solution at room temperature for 30 min. Subsequently, the cells were incubated with 100 μL of Hoechst 33342 (5 μg/mL) at room temperature for 30 min, followed by observation under a fluorescent microscope. The percentage of EdU-positive cells was calculated from five random fields in three wells.

### Flow cytometric analysis

BGC823 and SGC7901 cells transfected with si-DUXAP8 were harvested 48 h after transfection by trypsinization. After double staining with FITC-Annexin V and propidium iodide (PI) had been performed using the FITC Annexin V Apoptosis Detection Kit (BD Biosciences), in accordance with the manufacturer’s recommendations, the cells were analyzed by flow cytometry (FACScan®; BD Biosciences) with CellQuest software (BD Biosciences). Cells were classified into viable cells, dead cells, early apoptotic cells, and apoptotic cells, and then the relative ratio of early apoptotic cells was compared with that of the control transfectant for each experiment.

### TUNEL staining

The TUNEL assay was performed with an apoptosis detection kit (KeyGEN BioTECH, China), in accordance with the manufacturer’s instructions. Randomly selected fields without significant necrosis in 10 high-power fields were assessed for TUNEL-positive cells. The TUNEL index was calculated as the number of cells with green nuclei relative to the total number of cells.

### Western blot assay and antibodies

Cell protein lysates were separated by 10% sodium dodecyl sulfate polyacrylamide gel electrophoresis (SDS-PAGE), transferred to 0.22-μm NC membranes (Sigma), and incubated with specific antibodies. ECL chromogenic substrate was used for quantification by densitometry (Quantity One software; Bio-Rad). β-actin antibody was used as a control.

### Subcellular fractionation location

The separation of nuclear and cytosolic fractions was performed using the PARIS Kit (Life Technologies), in accordance with the manufacturer’s instructions.

### RIP assay

RIP assays were performed using the EZMagna RIP kit (Millipore, Billerica, MA, USA), following the manufacturer’s protocol. BGC823 and SGC7901 cells at 80–90% confluence were scraped off and then lysed in complete RIP lysis buffer. A total of 100 μl of whole-cell extract was incubated with RIP buffer containing magnetic beads conjugated with antibodies that recognize EZH2, SUZ12, or DNMT1, or with control IgG (Millipore) for 6 h at 4°C. After the beads had been washed with washing buffer, the complexes were incubated with 0.1% SDS/0.5 mg/ml Proteinase K (30 min at 55°C) to remove the proteins. The RNA concentration was measured using a NanoDrop (Thermo Scientific) and the RNA quality was assessed using a bioanalyzer (Agilent, Santa Clara, CA, USA). Furthermore, purified RNA was subjected to qRT-PCR analysis to demonstrate the presence of DUXAP8 and HOTAIR using specific primers.

### ChIP assay

ChIP assays were performed using the EZ-CHIP kit, in accordance with the manufacturer’s instructions (Millipore). EZH2 and SUZ12 antibodies were obtained from Abcam. Histone H3 trimethyl Lys 27 antibody was obtained from Millipore. The ChIP primer sequences are listed in Supplementary Table S2.

### Tumor formation assay in a nude mouse model

Female athymic BALB/c nude mice (4 weeks old) were maintained under pathogen-free conditions and manipulated in accordance with the protocols approved by the Shanghai Medical Experimental Animal Care Commission. SGC7901 cells were stably transfected with sh-DUXAP8 or empty vector, harvested from six-well cell culture plates, washed with PBS, and resuspended at a concentration of 1 × 10^8^ cells/ml. A total of 100 μL of suspended cells was subcutaneously injected into a single side of the posterior flank of each mouse. Tumor growth was examined every 3 days and tumor volumes were calculated using the following equation: V = 0.5 × D × d^2^ (V, volume; D, longest diameter; d, diameter perpendicular to the longest diameter). At 15 days postinjection, the mice were euthanized and the subcutaneous growth of each tumor was examined. This study was carried out in strict accordance with the recommendations of the Guide for the Care and Use of Laboratory Animals of the National Institutes of Health. The protocol was approved by the Committee on the Ethics of Animal Experiments of Nanjing Medical University.

### Immunohistochemical (IHC) analysis

The primary tumors were immunostained for Ki-67 as previously described [41].

### Statistical analysis

All statistical analyses were performed using SPSS 17.0 software (IBM, SPSS, USA). The significance of differences between groups was estimated by Student’s t-test, χ2 test or Wilcoxon test, as appropriate. PFS and OS rates were calculated by the Kaplan-Meier method with the log-rank test applied for comparison. P values less than 0.05 were considered significant.

## SUPPLEMENTARY MATERIALS FIGURES AND TABLES







## References

[1] Tan P, Yeoh KG (2015). Genetics and Molecular Pathogenesis of Gastric Adenocarcinoma. Gastroenterology.

[2] Wang XN, Liang H (2010). Some problems in the surgical treatment of gastric cancer. Chin J Cancer.

[3] Saka M, Morita S, Fukagawa T, Katai H (2011). Present and future status of gastric cancer surgery. Jpn J Clin Oncol.

[4] Shen L, Shan YS, Hu HM, Price TJ, Sirohi B, Yeh KH, Yang YH, Sano T, Yang HK, Zhang X, Park SR, Fujii M, Kang YK, Chen LT (2013). Management of gastric cancer in Asia: resource-stratified guidelines. Lancet Oncol.

[5] Zhang EB, Kong R, Yin DD, You LH, Sun M, Han L, Xu TP, Xia R, Yang JS, De W Chen J (2014). Long noncoding RNA ANRIL indicates a poor prognosis of gastric cancer and promotes tumor growth by epigenetically silencing of miR-99a/miR-449a. Oncotarget.

[6] Pei B, Sisu C, Frankish A, Howald C, Habegger L, Mu XJ, Harte R, Balasubramanian S, Tanzer A, Diekhans M, Reymond A, Hubbard TJ, Harrow J, Gerstein MB (2012). The GENCODE pseudogene resource. Genome Biol.

[7] Poliseno L, Salmena L, Zhang J, Carver B, Haveman WJ, Pandolfi PP (2010). A coding-independent function of gene and pseudogene mRNAs regulates tumour biology. Nature.

[8] Jacq C, Miller JR, Brownlee GG (1977). A pseudogene structure in 5S DNA of Xenopus laevis. Cell.

[9] Groen JN, Capraro D, Morris KV (2014). The emerging role of pseudogene expressed non-coding RNAs in cellular functions. Int J Biochem Cell Biol.

[10] Xiao-Jie L, Ai-Mei G, Li-Juan J, Jiang X (2015). Pseudogene in cancer: real functions and promising signature. J Med Genet.

[11] Kalyana-Sundaram S, Kumar-Sinha C, Shankar S, Robinson DR, Wu YM, Cao X, Asangani IA, Kothari V, Prensner JR, Lonigro RJ, Iyer MK, Barrette T, Shanmugam A (2012). Expressed pseudogenes in the transcriptional landscape of human cancers. Cell.

[12] Goodhead I, Darby AC (2015). Taking the pseudo out of pseudogenes. Curr Opin Microbiol.

[13] Jingsi T, Mingyao Y, Ying L (2015). Functional roles of pseudogenes in cancers. Yi Chuan.

[14] Kung JT, Colognori D, Lee JT (2013). Long noncoding RNAs: past, present, and future. Genetics.

[15] ENCODE Project Consortium (2012). An integrated encyclopedia of DNA elements in the human genome. Nature.

[16] Zhou M, Xu W, Yue X, Zhao H, Wang Z, Shi H, Cheng L, Sun J (2016). Relapse-related long non-coding RNA signature to improve prognosis prediction of lung adenocarcinoma. Oncotarget.

[17] Zhou M, Wang X, Shi H, Cheng L, Wang Z, Zhao H, Yang L, Sun J (2016). Characterization of long non-coding RNA-associated ceRNA network to reveal potential prognostic lncRNA biomarkers in human ovarian cancer. Oncotarget.

[18] Huang JL, Ren TY, Cao SW, Zheng SH, Hu XM, Hu YW, Lin L, Chen J, Zheng L, Wang Q (2015). HBx-related long non-coding RNA DBH-AS1 promotes cell proliferation and survival by activating MAPK signaling in hepatocellular carcinoma. Oncotarget.

[19] Hayashi H, Arao T, Togashi Y, Kato H, Fujita Y, De Velasco MA, Kimura H, Matsumoto K, Tanaka K, Okamoto I, Ito A, Yamada Y, Nakagawa K, Nishio K (2015). The OCT4 pseudogene POU5F1B is amplified and promotes an aggressive phenotype in gastric cancer. Oncogene.

[20] Mei D, Song H, Wang K, Lou Y, Sun W, Liu Z, Ding X, Guo J (2013). Up-regulation of SUMO1 pseudogene 3 (SUMO1P3) in gastric cancer and its clinical association. Med Oncol.

[21] Bosc DG, Graham KC, Saulnier RB, Zhang C, Prober D, Gietz RD, Litchfield DW (2000). Identification and characterization of CKIP-1, a novel pleckstrin homology domain-containing protein that interacts with protein kinase CK2. J Biol Chem.

[22] Tokuda E, Fujita N, Oh-hara T, Sato S, Kurata A, Katayama R, Itoh T, Takenawa T, Miyazono K, Tsuruo T (2007). Casein kinase 2-interacting protein-1, a novel Akt pleckstrin homology domain-interacting protein, down-regulates PI3K/Akt signaling and suppresses tumor growth in vivo. Cancer Res.

[23] Human gastric mucosa: 3 paired hTERT-negative para cancerous tissue (Control) vs. hTERT-positive gastric cancer tissue.

[24] Cho JY, Lim JY, Cheong JH, Park YY, Yoon SL, Kim SM, Kim SB, Kim H, Hong SW, Park YN, Noh SH, Park ES, Chu IN (2011). Gene expression signature-based prognostic risk score in gastric cancer. Clin Cancer Res.

[25] Khalil AM, Guttman M, Huarte M, Garber M, Raj A, Rivea MD, Thomas K, Presser A, Bernstein BE, van Oudenaarden A, Regev A, Lander ES, Rinn JL (2009). Many human large intergenic noncoding RNAs associate with chromatin-modifying complexes and affect gene expression. Proc Natl Acad Sci U S A.

[26] Marchese FP, Huarte M (2014). Long non-coding RNAs and chromatin modifiers: their place in the epigenetic code. Epigenetics-Us.

[27] Xie M, Sun M, Zhu YN, Xia R, Liu YW, Ding J, Ma HW, He XZ, Zhang ZH, Liu ZJ, Liu XH, De W (2015). Long noncoding RNA HOXA-AS2 promotes gastric cancer proliferation by epigenetically silencing P21/PLK3/DDIT3 expression. Oncotarget.

[28] Gupta RA, Shah N, Wang KC, Kim J, Horlings HM, Wong DJ, Tsai MC, Hung T, Argani P, Rinn JL, Wang Y, Brzoska P, Kong B (2010). Long non-coding RNA HOTAIR reprograms chromatin state to promote cancer metastasis. Nature.

[29] Ruan K, Fang X, Ouyang G (2009). MicroRNAs: novel regulators in the hallmarks of human cancer. Cancer Lett.

[30] Nagano T, Fraser P (2011). No-nonsense functions for long noncoding RNAs. Cell.

[31] Guo X, Deng L, Deng K, Wang H, Shan T, Zhou H, Liang Z, Xia J, Li C (2015). Pseudogene PTENP1 Suppresses Gastric Cancer Progression by Modulating PTEN. Anticancer Agents Med Chem.

[32] Grander D, Johnsson P (2015). Pseudogene-Expressed RNAs: Emerging Roles in Gene Regulation and Disease. Curr Top Microbiol Immunol.

[33] Chan WL, Chang JG (2014). Pseudogene-derived endogenous siRNAs and their function. Methods Mol Biol.

[34] Tay Y, Kats L, Salmena L, Weiss D, Tan SM, Ala U, Karreth F, Poliseno L, Provero P, Di Cunto F, Lieberman J, Rigoutsos I, Pandolfi PP (2011). Coding-independent regulation of the tumor suppressor PTEN by competing endogenous mRNAs. Cell.

[35] Yu G, Yao W, Gumireddy K, Li A, Wang J, Xiao W, Chen K, Xiao H, Li H, Tang K, Ye Z, Huang Q, Xu H (2014). Pseudogene PTENP1 functions as a competing endogenous RNA to suppress clear-cell renal cell carcinoma progression. Mol Cancer Ther.

[36] Chan WL, Yuo CY, Yang WK, Hung SY, Chang YS, Chiu CC, Yeh KT, Huang HD, Chang JG (2013). Transcribed pseudogene psiPPM1K generates endogenous siRNA to suppress oncogenic cell growth in hepatocellular carcinoma. Nucleic Acids Res.

[37] Hawkins PG, Morris KV (2010). Transcriptional regulation of Oct4 by a long non-coding RNA antisense to Oct4-pseudogene 5. Transcription.

[38] Varambally S, Cao Q, Mani RS, Shankar S, Wang X, Ateeq B, Laxman B, Cao X, Jing X, Ramnarayanan K, Brenner JC, Yu J, Kim JH (2008). Genomic loss of microRNA-101 leads to overexpression of histone methyltransferase EZH2 in cancer. Science.

[39] Ning X, Shi Z, Liu X, Zhang A, Han L, Jiang K, Kang C, Zhang Q (2015). DNMT1 and EZH2 mediated methylation silences the microRNA-200b/a/429 gene and promotes tumor progression. Cancer Lett.

[40] Nie J, Liu L, Xing G, Zhang M, Wei R, Guo M, Li X, Xie P, Li L, He F, Han W, Zhang L (2014). CKIP-1 acts as a colonic tumor suppressor by repressing oncogenic Smurf1 synthesis and promoting Smurf1 autodegradation. Oncogene.

[41] Liao WT, Wang X, Xu LH, Kong QL, Yu CP, Li MZ, Shi L, Zeng MS, Song LB (2009). Centromere protein H is a novel prognostic marker for human nonsmall cell lung cancer progression and overall patient survival. Cancer-Am Cancer Soc.

