# Supplementary material for: The pseudogene derived long noncoding RNA DUXAP8 promotes gastric cancer cell proliferation and migration via epigenetically silencing PLEKHO1 expression
Source: Oncotarget. 2016 Aug 5;8(32):52211–24. doi: 10.18632/oncotarget.11075 (PMC5581023; doi:10.18632/oncotarget.11075)
Supplement: Supplementary file 1 [file oncotarget-08-52211-s001.pdf]

# The pseudogene derived long noncoding RNA DUXAP8 promotes gastric cancer cell proliferation and migration via epigenetically silencing PLEKHO1 expression

## SUPPLEMENTARY FIGURES

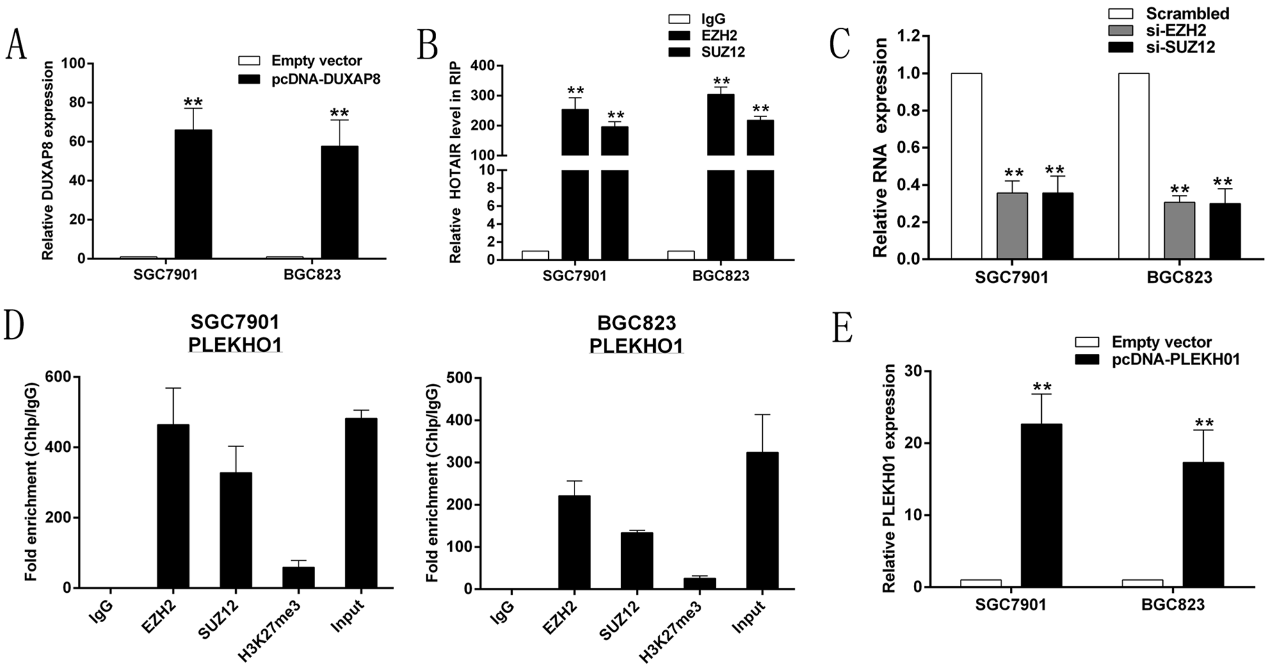


**Supplementary Figure S1: A.** QRT-PCR analysis of DUXAP8 expression in pcDNA-DUXAP8 transfected GC cells **B.** RIP experiments were performed in SGC7901, BGC823 cells and the coprecipitated RNA was subjected to qRT-PCR for HOTAIR. The fold enrichment of HOTAIR in EZH2/SUZ12/LSD1 RIP is relative to its matching IgG control. **C.** QRT-PCR analysIs of EZH2 and SUZ12 expression levels in control, si-EZH2 and si-SUZ12 treated GC cells. **D.** ChIp-qRT-PCR of EZH2 occupancy, SUZ12 occupancy and H3K27me3 binding in the PLEKHO1 promoters in GC cells. **E.** QRT-PCR analysis of PLEKHO1 expression in pcDNA-PLEKHO1-transfected GC cells. Error bars indicate mean ± standard errors of the mean. $*P < 0.05$, $**P < 0.01$.

**Supplementary Table S1:**

**See Supplementary File 1**

**Supplementary Table S2:**

**See Supplementary File 2**
