# Supplementary material for: The pseudogene derived long noncoding RNA DUXAP8 promotes gastric cancer cell proliferation and migration via epigenetically silencing PLEKHO1 expression
Source: Oncotarget. 2016 Aug 5;8(32):52211–24. doi: 10.18632/oncotarget.11075 (PMC5581023; doi:10.18632/oncotarget.11075)
Supplement: Supplementary file 3 [file oncotarget-08-52211-s003.doc]

**Primers for qPCR**

GAPDH F GGGAGCCAAAAGGGTCAT

GAPDH R GAGTCCTTCCACGATACCAA

DUXAP8 F GAGAAGCAGTGGTGGGTTCC

DUXAP8 R GAGCAACACAGATGAACCGC

PLEKHO1 F GGGACCAGCTCTACATCTCTG

PLEKHO1 R TGGAGTGGGCAAGAGTAAACT

DPM3 F CTTTGGGGACTAGCGATCCTG

DPM3 R GCCTCCTGTATCTGGCTCTG

HABP4 F GAAGCGGACTCCTAGAAGAGG

HABP4 R CATAGGGTCGGTATTCCCTGTA

RBMS3 F TGGACCATCCCATGTCAATGC

RBMS3 R CCAACGAAAGGTGATTCATCTGC

RARRES1 F CTGGGATTTGGCTTTCCTTGG

RARRES1 R GGTTTTTCTTACCCACTGCCTC

HNRNPM F GGGTGAAGGAGAACGACCTG

HNRNPM R ACAACAGCACATCCCTTTTTCT

PLEKHO1 PF TGTCCAGCAAGCCCTCAAAA

PLEKHO1 PR CCCAAACCATCTTGTCCCCT

**siRNA sequence**

si-DUXAP8 1# UUUAGACCCAUUCUCGUAUGGAGGU ACCUCCAUACGAGAAUGGGUCUAAA

si-DUXAP8 2# CAGCAUACUUCAAAUUCACAGCAAA UUUGCUGUGAAUUUGAAGUAUGCUG

si-DUXAP8 3# UUUAGACCCAUUCUCGUAUGGAGGU ACCUCCAUACGAGAAUGGGUCUAAA
